# The role of tumor microenvironment in resistance to anti-angiogenic therapy

**DOI:** 10.12688/f1000research.11771.1

**Published:** 2018-03-15

**Authors:** Shaolin Ma, Sunila Pradeep, Wei Hu, Dikai Zhang, Robert Coleman, Anil Sood

**Affiliations:** 1 Department of Gynecologic Oncology and Reproductive Medicine, The University of Texas MD Anderson Cancer Center, Houston, TX, USA; 2 Reproductive Medicine Research Center, Department of Gynecology and Obstetrics, The Sixth Affiliated Hospital, Sun Yat-sen University, Guangzhou, Guangdong province, China; 3 Department of Cancer Biology, The University of Texas MD Anderson Cancer Center, Houston, TX, USA; 4 Center for RNA Interference and Non-Coding RNA, The University of Texas MD Anderson Cancer Center, Houston, TX, USA

**Keywords:** tumor microenvironment, anti-angiogenic therapy, drug resistance, MET signaling

## Abstract

Anti-angiogenic therapy has been demonstrated to increase progression-free survival in patients with many different solid cancers. Unfortunately, the benefit in overall survival is modest and the rapid emergence of drug resistance is a significant clinical problem. Over the last decade, several mechanisms have been identified to decipher the emergence of resistance. There is a multitude of changes within the tumor microenvironment (TME) in response to anti-angiogenic therapy that offers new therapeutic opportunities. In this review, we compile results from contemporary studies related to adaptive changes in the TME in the development of resistance to anti-angiogenic therapy. These include preclinical models of emerging resistance, dynamic changes in hypoxia signaling and stromal cells during treatment, and novel strategies to overcome resistance by targeting the TME.

## Introduction

**Figure 1.**
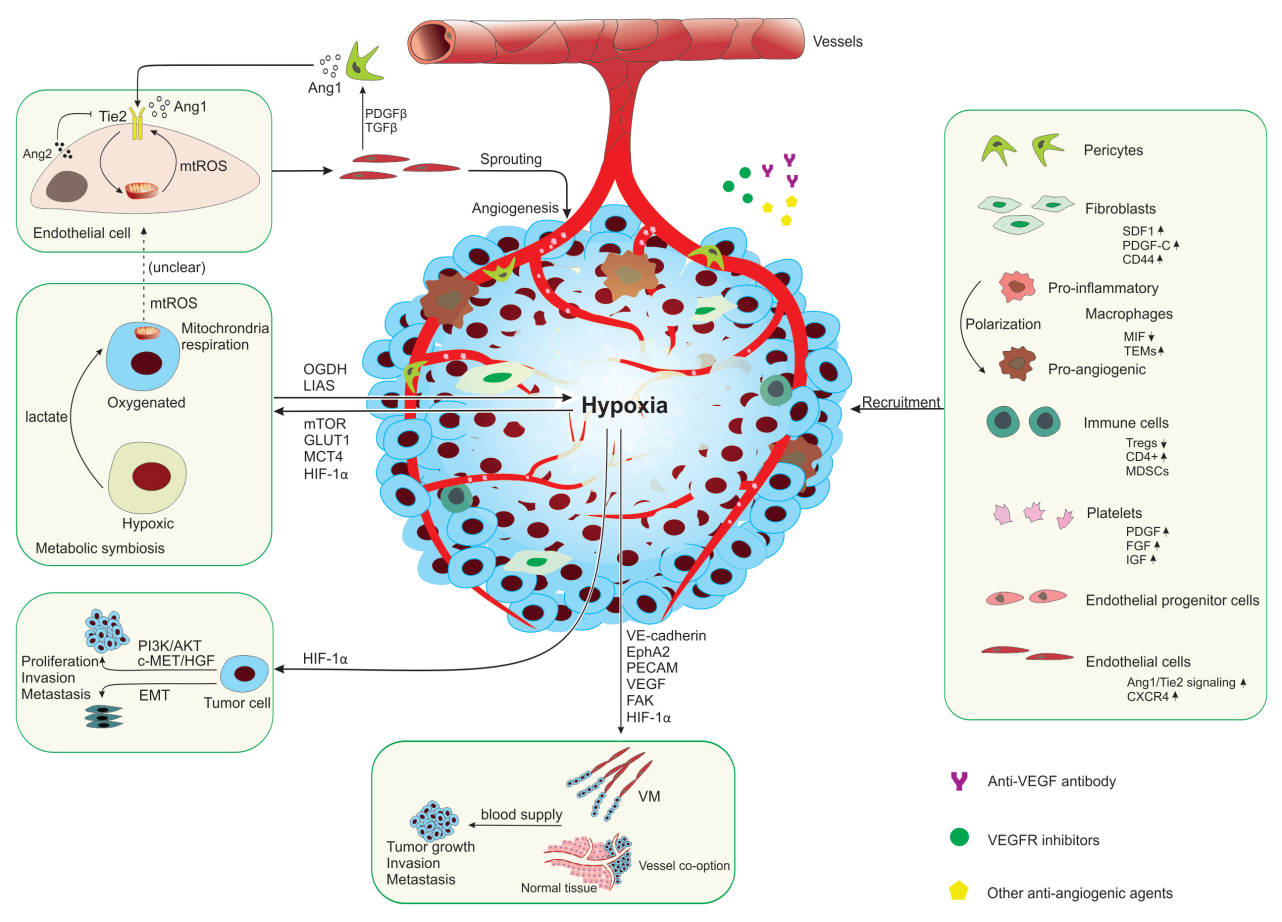
Schematic illustration of the role of the tumor microenvironment in resistance to anti-angiogenic therapy. Anti-angiogenic therapy inhibits tumor growth by reducing vessel density; however, the subsequent hypoxia and the responsive genes can cause resistance to such therapy. The hypoxia-related metabolic symbiosis, invasion and metastasis, vessel co-option, and vasculogenic mimicry (VM) lead to resistance to anti-angiogenic therapy. The recruitment of stromal cells also plays a critical role in resistance to anti-angiogenic therapy. Ang1/2, angiopoietin 1/2; CXCR4, C-X-C chemokine receptor type 4; EMT, epithelial-to-mesenchymal transition; EphA2, Eph receptor A2; FAK, focal adhesion kinase; FGF, fibroblast growth factor; GLUT1, glucose transporter-1; HGF, hepatocyte growth factor; HIF-1α, hypoxia-inducible factor 1α; IGF, insulin-like growth factor; LIAS, lipoic acid synthase; MCT4, monocarboxylate transporter 4; MDSC, myeloid-derived suppressor cell; MIF, macrophage migration inhibitory factor; mTOR, mammalian target of rapamycin; mtROS, mitochondria reactive oxygen species; OGDH, oxoglutarate dehydrogenase; PDGF, platelet-derived growth factor; PECAM, platelet endothelial cell adhesion molecule; SDF1, stromal cell-derived factor 1; TEM; Ties-expressing macrophage; Treg, regulatory T cell; VE-cadherin, vascular endothelial cadherin; VEGF, vascular endothelial growth factor.

Angiogenesis is well recognized as an important step in the growth and progression of many tumor types ^1^. Over the last 15 years, anti-angiogenic therapy has become an effective modality for cancer therapy. Several vascular endothelial growth factor/receptor (VEGF/R) inhibitors have been approved by the US Food and Drug Administration for various solid tumors, including metastatic colorectal cancer (mCRC), metastatic renal cell cancer, metastatic gastric cancer, non-small-cell lung cancer, recurrent/metastatic cervical cancer, recurrent ovarian cancer, and glioblastoma multiforme (GBM). Although improvements in objective response and progression-free survival (PFS) have been seen, the impact of anti-angiogenic therapy on patient overall survival (OS) is limited ( Table 1) because of a host of factors, including the induction of resistance ^2^. The modes of resistance to angiogenesis inhibitors, mechanisms of acquired or intrinsic resistance, and strategies for overcoming resistance have been discussed (see 3– 5). Meanwhile, new mechanisms and therapies for anti-angiogenic resistance have emerged over the last 3–5 years. Evidence suggests that changes in the tumor microenvironment (TME) play a critical role in such adaptation ^6^. This review focuses mainly on the role of the TME in response and resistance to anti-angiogenic therapy ( Figure 1), and novel strategies to overcome resistance by targeting the TME are also discussed.

**Table 1.** Survival data of phase II/III clinical trials with anti-angiogenic therapy in last 3 years.

Tumor type	Study regimen	Number of enrolled patients	Target	PFS	OS	Phase	Main finding	Reference
Platinum- sensitive recurrent ovarian cancer	Paclitaxel and carboplatin versus plus bevacizumab	674	VEGF	10.4: 13.8 months HR = 0.63 95% CI 0.53–0.74 *p* <0.0001	37.3: 42.2 months HR = 0.83 95% CI 0.68–1.01 *P* = 0.056	III	Adding bevacizumab to chemotherapy prolongs OS, but there is no statistical significance	22
Platinum- sensitive/ resistant recurrent ovarian cancer	Pegylated liposomal doxorubicin plus placebo versus plus trebananib	223	Ang-1 Ang-2	7.2: 7.6 months HR = 0.92 95% CI 0.68–1.24 *p* = 0.57	17.0: 19.4 months HR = 0.94 95% CI 0.64–1.39 *p* = 0.76	III	Trebananib demonstrates anti-cancer activity but does not improve PFS or OS	23
Recurrent ovarian cancer	Paclitaxel plus placebo versus plus trebananib	461	Ang-1 Ang-2	5.4: 7.2 months HR = 0.66 95% CI 0.57–0.77 *p* <0.0001	17.3: 19.0 months HR = 0.86 95% CI 0.69–1.08 *p* = 0.19	III	Adding trebananib to paclitaxel improves PFS	24
Platinum- sensitive recurrent ovarian cancer	Gemcitabine and carboplatin alone versus plus bevacizumab	484	VEGF	8.4: 12.4 months HR = 0.48 95% CI 0.39–0.61 Long-rank *p* <0.0001	32.9: 33.6 months HR = 0.95 95% CI 0.77–1.18 Long-rank *p* = 0.65	III	Adding bevacizumab to chemotherapy statistically significantly improves PFS	25, 26
Platinum- resistant recurrent ovarian cancer	Paclitaxel/topotecan/pegylated liposomal doxorubicin alone versus plus bevacizumab	361	VEGF	3.4: 6.7 months HR = 0.48 95% CI 0.38–0.60 *p* <0.001	13.3: 16.6 months HR = 0.85 95% CI 0.66–1.08 *p* <0.174	III	Adding bevacizumab to chemotherapy statistically significantly improves PFS	27
Newly diagnosed ovarian cancer	Carboplatin/paclitaxel alone versus plus bevacizumab	1,528	VEGF	16.3: 19.4 months HR = 0.93 95% CI 0.83–1.05 Long-rank *p* = 0.25	48.6: 48.8 months HR = 0.99 95% CI 0.85–1.14 Long-rank *p* = 0.85	III	Adding bevacizumab to platinum-based therapy does not improve OS	28
Relapsed platinum- sensitive ovarian cancer	Placebo alongside chemotherapy and then placebo only maintenance versus cediranib alongside chemotherapy then cediranib maintenance	486	VEGFR1–3	8.7: 11.0 months HR = 0.56 95% CI 0.44–0.72 *p* <0.0001	21.0: 26.3 months HR = 0.77 95% CI 0.55–1.07 *p* = 0.11	III	Adding cediranib to chemotherapy and continued as maintenance significantly improves PFS	29
Recurrent ovarian, tubal, or peritoneal carcinoma	Bevacizumab versus bevacizumab plus fosbretabulin	107	VEGF	4.8: 7.3 months HR = 0.69 90% CI 0.47–1.00 *p* = 0.05	22.0: 24.6 months HR = 0.85 90% CI 0.54–1.34 *p* value not provided	II	Adding fosbretabulin to bevacizumab may enhance its efficacy	30
Advanced ovarian cancer	Carboplatin and paclitaxel plus placebo versus plus nintedanib	1,366	VEGFR, PDGFR, and FGFR	16.6: 17.2 months HR = 0.84 95% CI 0.72–0.98 *p* = 0.024	Pending	III	Nintedanib in combination with carboplatin and paclitaxel significantly improves PFS	31
Advanced cervical cancer	Cisplatin plus paclitaxel/topotecan plus paclitaxel with or without bevacizumab	452	VEGF	8.2: 5.9 months HR = 0.67 95% CI 0.54–0.82 *p* = 0.002	17.0: 13.3 months HR = 0.71 98% CI 0.54–0.95 *p* = 0.004	III	Addition of bevacizumab to combination chemotherapy can improve PFS and OS	32
Metastatic or recurrent cervical cancer	Carboplatin and paclitaxel plus placebo versus plus cediranib	69	VEGFR1–3	6.7: 8.1 months HR = 0.58 80% CI 0.40–0.85 *p* = 0.032	14.8: 13.6 months HR = 0.94 80% CI 0.65–1.36 *p* = 0.42	II	Adding cediranib to carboplatin and paclitaxel improves PFS	33
Advanced non-squamous non-small-cell lung cancer	Bevacizumab plus docetaxel versus docetaxel alone	100	VEGF	4.4: 3.4 months HR = 0.71 95% CI 0.47–1.09 *p* = 0.058	13.1: 11.0 months HR = 0.74 95% CI 0.46–1.19 *p* = 0.11	II	Adding bevacizumab to docetaxel improves PFS	34
Advanced or recurrent non-squamous non-small-cell lung cancer	Carboplatin/paclitaxel alone versus plus bevacizumab	276	VEGF	6.5: 9.2 months HR = 0.40 95% CI 0.29–0.54 *p* <0.001	17.7: 24.3 months HR = 0.68 95% CI 0.50–0.93 *p* = 0.0154	III	Adding bevacizumab to carboplatin/paclitaxel is well tolerated and shows a treatment benefit	35
Untreated extensive small- cell lung cancer	Chemotherapy alone versus plus bevacizumab	147	VEGF	5.5: 5.3 months HR = 1.1 95% CI 0.7–1.7 unadjusted *p* = 0.82	13.3: 11.1 months HR = 0.8 95% CI 0.5–1.3 unadjusted *p* = 0.35	II–III	Adding bevacizumab to chemotherapy does not improve PFS or OS	36
Metastatic colorectal cancer	Bevacizumab versus bevacizumab plus erlotinib	700	VEGF, VEGFR	4.9: 5.4 months stratified HR = 0.81 95% CI 0.66–1.01 *p* = 0.059 unstratified HR = 0.78 95% CI 0.68–0.96 *p* = 0.019	24.9: 22.1 months stratified HR = 0.79 95% CI 0.63–0.99 *p* = 0.036 unstratified HR = 0.79 95% CI 0.64–0.98 *p* = 0.035	III	Maintenance therapy with erlotinib plus bevacizumab shows signs of greater activity than bevacizumab alone	37
Metastatic colorectal carcinoma	Ramucirumab versus placebo	1,072	VEGFR2	5.7: 4.5 months HR = 0.793 95% CI 0.70–0.90 *p* = 0.0005	13.3: 11.7 months HR = 0.844 95% CI 0.73–0.98 *p* = 0.0219	III	Adding ramucirumab to FOLFIRI significantly improves PFS and OS	38
Advanced breast cancer	Letrozole or fulvestrant alone versus plus bevacizumab	374	VEGF	14.4: 19.3 months HR = 0.83 95% CI 0.65–1.06 *p* = 0.126	51.8: 52.1 months HR = 0.87 95% CI 0.58–1.32 *p* = 0.518	III	Adding bevacizumab to gemcitabine-docetaxel fails to improve PFS or OS	39
Glioblastoma	Bevacizumab and temozolomide versus temozolomide alone	93	VEGF	4.8: 2.2 months; HR = 0.70, 95% CI 0.46–1.07 *p* = 0.10	10.6: 7.7 months HR = 0.68 95% CI 0.44–1.04 *p* = 0.07	II	Adding bevacizumab to temozolomide is more active than temozolomide alone	40
Non-metastatic renal cell carcinoma	Sunitinib or sorafenib versus placebo	1,323	VEGF, PDGFR, Ras-Raf- MAPK	(Sunitinib: placebo) 70: 79.6 months HR = 1.02 97.5% CI 0.85–1.23 *p* = 0.8038; (sorafenib: placebo) HR = 0.97 97.5% CI 0.80–1.17 Stratified log-rank *p* = 0.7184	(Sunitinib: placebo) OS not reached HR = 1.17 97.5% CI 0.90–1.52 *p* = 0.1762 (sorafenib: placebo) HR = 0.98 97.5% CI 0.75–1.28 Stratified log-rank *p* = 0.8577	III	Adjuvant treatment with sorafenib or sunitinib shows no survival benefit	41
Metastatic renal cell carcinoma	Cabozantinib versus sunitinib	157	VEGFR2 MET AXL	8.2: 5.6 months HR = 0.66 95% CI 0.46–0.95 *p* = 0.012	30.3: 21.8 months HR = 0.80 95% CI 0.50–1.26 *p* value not provided	II	Cabozantinib demonstrated a better clinical benefit than sunitinib	42
Advanced renal cell carcinoma	Cabozantinib versus everolimus	658	VEGFR2 MET AXL mTOR	7.4: 3.9 months HR = 0.51 95% CI 0.41–0.62 *p* <0.0001	21.4: 16.5 months HR = 0.66 95% CI 0.53–0.83 *p* = 0.00026	III	Cabozantinib increases PFS, OS, and objective response compared with everolimus	43
Metastatic melanoma	Cabozantinib versus placebo	77	VEGFR2 MET AXL	4.1: 2.8 months HR = 0.59 95% CI Not reached *p* = 0.284	Not provided	II	Cabozantinib has clinical anti-tumor activity but needs further clinical investigation	44
Pleural mesothelioma	Pemetrexed plus cisplatin with or without bevacizumab	448	VEGF	9.2: 7.3 months HR = 0.61 95% CI 0.50–0.75 *p* <0.0001	18.8: 16.1 months HR = 0.77 95% CI 0.62–0.95 *p* = 0.0167	III	Adding bevacizumab to pemetrexed plus cisplatin significantly improves PFS and OS	45
Metastatic uterine leiomyosarcoma	Gemcitabine-docetaxel plus placebo versus plus bevacizumab	107	VEGF	6.2: 4.2 months HR = 1.12 95% CI 0.74–1.7 *p* = 0.58	26.9: 23.3 months HR = 1.07 95% CI 0.63–1.81 *p* = 0.81	III	Adding bevacizumab to gemcitabine-docetaxel fails to improve PFS or OS	46
Metastatic soft tissue sarcoma	Pazopanib versus placebo	47	c-KIT, FGFR, PDGFR, VEGFR	24.7: 7.0 weeks HR = 0.41 95% CI 0.19–0.90 *p* = 0.002	15.4: 14.9 months HR = 0.87 95% CI 0.41–1.83 *p* = 0.687	III	Pazopanib significantly improves PFS	47

Ang, angiopoietin; CI, confidence interval; FGFR, fibroblast growth factor receptor; FOLFIRI, folinic acid, fluorouracil, and irinotecan; HR, hazard ratio; OS, overall survival; MAPK, mitogen-activated protein kinase; mTOR, mammalian target of rapamycin; PDGFR, platelet-derived growth factor receptor; PFS, progression-free survival; VEGF, vascular endothelial growth factor; VEGFR, vascular endothelial growth factor receptor.

## The role of hypoxia in resistance to anti-angiogenic therapy

Previous studies have shown that resistance to anti-angiogenic therapy is associated with hypoxia-induced alterations, VEGF-independent cytokine-driven endothelial growth, mobilization of bone marrow-derived pro-angiogenic hematopoietic cells or endothelial progenitors, and vessel co-option ^2– 5, 7^. Anti-angiogenic therapy inhibits tumor growth effectively by reducing vessel density; however, the subsequent expression of hypoxia-inducible factors (HIFs) and the responsive genes (for example, *VEGF*, *VEGFR*, *carbonic anhydrase* [ *CA*] *IX*, and *CAXII*) can lead to therapeutic resistance ^8^. In recent years, there has been growing evidence that hypoxia-triggered overexpression of HIF subunits and the activated downstream pathways play a critical role in resistance to anti-angiogenic therapy.

### Role of HIF-1α in anti-angiogenic therapy

There are three α subunits (HIF-1α, -2α, and -3α) and one β subunit in the HIF family. HIF-1α is the oxygen-regulated subunit that has been studied in inflammation, diabetes, cardiovascular disease, and cancer. In the presence of O _2_, prolyl hydroxylase domain (PHD) proteins (principally PHD2) can use O _2_ and a-ketoglutarate to subject HIF-1α to prolyl hydroxylation on proline residue 402/564 ^9^. Von Hippel–Lindau protein recruits ubiquitin ligase complex by interacting with Elogin C after HIF-1α prolyl hydroxylation. Then, ubiquitinated HIF-1α can be recognized and degraded by the proteasome. Meanwhile, factor inhibiting 1 (FIH-1), which is an asparaginyl hydroxylase, can block HIF-1α transcription by blocking the interaction of HIF-1α transactivation domain with its co-activators p300 and CBP ^9^. When O _2_ is deprived, the inhibition of prolyl hydroxylase (for example, PHD2) and asparaginyl hydroxylase activity (for example, FIH-1) increases the stability and transcription of HIF-1α and consequently causes the dimerization of HIF-1α and HIF-1β to form HIF1. HIF1 can bind to target genes and increase gene transcription ^9^.

HIF-1α is a potent pro-angiogenic factor that has been associated with the regulation of VEGF, stromal cell-derived factor 1 (SDF1), plasminogen activator inhibitor 1 (PAI1), angiopoietins (Ang-1 and -2), platelet-derived growth factor (PDGF), Tie2 receptor, and matrix metalloproteinases (MMP-2 and -9) ^10, 11^. The expression of HIF-1α is driven by hypoxia and mediated by histone deacetylase (HDAC). Deacetylation by HDAC is a critical post-translational modification to HIF-1α signaling. Upregulation of HDACs has been observed in response to increasing HIF-1α signaling under hypoxia ^12^. A phase I clinical trial showed that the addition of HDAC inhibitor abexinostat to pazopanib led to a durable response in some patients who experienced progression during anti-VEGF therapy ^13^. In addition, inhibiting HDACs can abrogate the expression of HIF-1α protein in hypoxic conditions and there is an additive or synergistic effect between HDAC and VEGFR inhibitors in resistant cancers ^12, 14^. *In vitro* and *in vivo* data have demonstrated that nucleus accumbens-associated protein-1 (NAC1), a critical molecule in promoting glycolysis under hypoxia, mediates glycolysis via HDAC4-mediated stabilization of HIF-1α. The knockdown of NAC1 exhibits anti-tumor effects of bevacizumab, which means that NAC1 may be involved in resistance to anti-angiogenic therapy ^15^. Thus, NAC1-HDAC4-HIF-1α signaling might be an important pathway in regulating resistance under hypoxia.

### MET signaling

HIF-1α can also regulate the c-MET/HGF pathway, which can induce tumor angiogenesis through stimulation of endothelial cell (EC) proliferation, migration, and tubulogenesis ^16^. Hypoxia enhances c-MET/HGF signaling by activating HIF-1α in several types of cancers such as lung, ovarian, and cervical cancers ^17^. MET and VEGFR pathways share common downstream molecules such as mitogen-activated protein kinase (MAPK), ERK, AKT, and focal adhesion kinase (FAK), and the activation of c-MET/HGF might lead to the activation of VEGFR signaling. It has been shown that MET enhances the expression of VEGFA by interacting with src homology 2 domain containing and suppressing angiogenesis suppressor thrombospondin1 ^18^. Other studies have also demonstrated that MET contributes to resistance to VEGF(R) inhibitors via the activation of ERK–MAPK and PI3K–AKT signaling ^19^. To identify mediators of resistance to anti-angiogenic therapy, Jahangiri *et al*. ^20^ generated a novel glioma cell-derived bevacizumab-resistant xenograft model by injecting cells subcutaneously and harvesting the least responsive xenograft tumor cells and implanting them into mice with long-term treatment of bevacizumab (10 mg/kg). After the tumors were serially passaged subcutaneously (three cycles) *in vivo*, a stably resistant xenograft model was developed ^20^. Microarray analysis of this model showed upregulation of c-Met; adding a MET inhibitor with bevacizumab treatment impeded tumor invasion and prolonged survival in resistant mice ^20^. Cabozantinib, a multi-targeting inhibitor of MET, VEGFR2, AXL, and RET, can overcome HGF/MET signaling-mediated resistance to pan-VEGFR inhibition in neuroblastoma mouse models ^21^. Furthermore, c-MET can form a complex with β1-integrin extensively in bevacizumab-resistant GBM and result in increased migration ^48^. It has been demonstrated in some preclinical studies that increased invasiveness and metastasis is caused by c-MET activation due to the inhibition of VEGF signaling, which also results in resistance to anti-angiogenic therapy.

c-MET/HGF signaling leads to the activation of numerous signaling cascades, especially those related to epithelial-to-mesenchymal transition (EMT). Anti-angiogenic treatment can activate the EMT repressor ZEB2 by upregulating HIF-1α. ZEB2 can downregulate ephrinB2 through promoter binding to enhance tumor invasiveness ^49^. The development of EMT has been confirmed in a multi-generational glioblastoma xenograft model, which is established by selecting the fastest growing tumor during bevacizumab treatment in each generation and reimplanting them into new mice. The authors observed that critical EMT transcription factors SNAI2 and ZEB2 were upregulated during bevacizumab treatment ^50^. A recent study showed a synergistic effect of c-MET and VEGFR inhibitor (sunitinib) in reducing invasiveness and metastasis of RIP-Tag2 and Panc-1 tumors ^51^. Similarly, another study demonstrated that VEGF could directly and negatively regulate GBM invasion by inhibiting MET activation, which is dependent on VEGFR2 ^52^. Consequently, the broad use of anti-angiogenic therapy could restore and increase MET levels and induce EMT, which is confirmed in GBM patients who are resistant to bevacizumab ^52^. Overall, these data suggest that HGF/MET signaling plays a crucial role in increased invasiveness, metastasis, and drug resistance during anti-angiogenic therapy. The phase III METEOR trial demonstrated that cabozantinib (MET, VEGFR, and AXL inhibitor) treatment resulted in improvements in PFS, OS, and objective response rate in patients with advanced renal cell cancer and bone metastases after previous VEGFR inhibition therapy ^53^. Similarly, a phase II study showed that the dual MET/VEGFR2 inhibitor foretinib had anti-tumor activity in patients with papillary renal carcinoma and a high response rate in patients with germline *MET* mutations ^54^. These studies suggest a promising future for combining MET and VEGF/R inhibitors to overcome drug resistance.

### Vascular mimicry

Tumor cells have a complex vasculature system that can develop compensatory mechanisms to evade therapeutic effect, such as revascularization. Vasculogenic mimicry (VM) is a blood supply system whereby vascular-like channels may form independently of ECs ^55^. VM is regulated by various molecules, including vascular endothelial cadherin (VE-cadherin), ephrin type-A receptor 2 (EphA2), platelet EC adhesion molecule (PECAM), VEGF, and FAK ^56^. In addition, hypoxia-related pathways, especially HIF-1α, are important regulatory mechanisms in the process of VM ^57^. Growing evidence indicates that tumor cells are capable of mimicking EC characteristics to form VM. It is reported that the VEGFR2 inhibitor sunitinib can increase VM under hypoxia by transforming tumor cells into endothelial-like cells ^58^. Another study showed that PECAM1 (also known as CD31, a mediator of angiogenesis that regulates EC–cell interactions) positive melanoma cells have the ability to form tube-like structures *in vitro* and could incorporate with vascular lumens *in vivo* ^59, 60^. It was also confirmed that PECAM1 ^+^ melanoma cells are enriched and might lead to resistance during anti-VEGF therapy ^60^. Similarly, in a breast cancer mouse model, VM channels were increased after treatment with sunitinib and related to increased hypoxia. However, this vessel regrowth exists only in the models bearing cells with the ability to form VM ^61^. Those findings showed that the process of VM depends mainly on specific tumor cell characteristics that can resemble EC features. Anti-angiogenic therapy-induced VM is highly related to hypoxia and leads to angiogenic rebound by forming endothelium-independent vascular channels.

### Blood vessel co-option

In addition to VM, cancer cells can develop another vascular network for the resupply of oxygen and nutrients to escape anti-angiogenic therapy by blood vessel co-option ^62^. Vessel co-option is a process whereby cancer cells “hijack” pre-existing vasculature and migrate along the vessels of host organs to gain a blood supply ^63^. It has been shown that vessel co-option occurs mainly in well-vascularized organs such as the brain, lungs, and liver in both human cancers and animal models of cancer ^63, 64^. A more recent study revealed that vessel co-option also occurs in lymph node metastases where it supports the growth of lymph node metastatic lesions. Furthermore, clinical evidence suggests that anti-angiogenic therapy (for example, bevacizumab) may not reduce vessel density in lymph node metastases from patients who received bevacizumab treatment ^65^. Other studies in preclinical models of glioblastoma and melanoma brain metastases have shown that tumor progression during treatment with anti-angiogenic drugs is associated with the induction of vessel co-option, which results in therapy resistance ^66, 67^. In human lung metastases from breast, colorectal, or renal cancer, vessel co-option appears to be common, and in preclinical models of lung metastasis, vessel co-option was shown to be associated with resistance to sunitinib. The predominant mechanism of vessel co-option in metastatic tumors in the lungs is a process whereby cancer cells invade alveolar spaces and co-opt alveolar walls and their constituent alveolar capillaries. Subsequently, pneumocytes are lost from these co-opted alveolar walls, leaving behind the co-opted alveolar capillaries ^68, 69^. Another preclinical study revealed that the VEGFR inhibitor sorafenib induces vessel co-option in an orthotopic model of hepatocellular carcinoma (HCC) and that this increase in vessel co-option was associated with resistance to sorafenib ^70^. Several pro-EMT transcription factors (for example, vimentin, ZEB1, and ZEB2) were upregulated significantly in the sorafenib-resistant tumors, which suggested a link between EMT and vessel co-option ^70^. Also, CD34 ^+^ microvessels and α-smooth muscle actin (αSMA) ^+^ pericytes were depleted in both sorafenib-sensitive and -resistant tumor tissues compared with tissues without treatment, which indicates that the acquired resistance was not induced by the re-induction of angiogenesis ^70^. Importantly, other studies have also demonstrated that anti-angiogenic therapy can promote cancer cell invasion and induce an EMT switch, which is linked to acquired resistance ^71, 72^. However, the molecular pathways involved in the induction of vessel co-option during anti-angiogenic therapy remain unclear. In addition, one study has shown that mCRC with histopathological features of co-opted vessels is associated with worse response to bevacizumab than patients with angiogenic metastases ^73^. However, further studies using patient samples obtained after treatment with anti-angiogenic therapy are needed to fully clarify the clinical association between vessel co-option and resistance to anti-angiogenic therapy.

### Metabolic symbiosis

Another compensatory mechanism to hypoxia is metabolic symbiosis, a process in which tumor cells in the oxygenated region can use lactate from hypoxic, glycolytic tumor cells to produce ATP ^74^. This metabolic shift is driven by HIF-1α and is associated with the activation of glycolytic genes ^75^. Pisarsky *et al*. ^76^ established a mouse orthotopic model with a stable murine breast cancer cell line (Py2T) and developed an evasive resistance model with long-term treatment with nintedanib (potent inhibitor of fibroblast growth factor [FGF] receptor 1 [FGFR1], 2, and 3, PDGF receptor α/β, and VEGFR1, 2, and 3). In this model, evasive resistance was found to be associated with the establishment of metabolic symbiosis but not tumor revascularization ^74, 76^. Allen *et al*. observed similar metabolic symbiosis with anti-angiogenic therapy in the RIP1-Tag2 transgenic mouse pancreatic neuroendocrine tumor (PanNET) model ^77^. Upregulation of glucose transporter 1 (GLUT1) and monocarboxylate transporter 4 (MCT4) in the hypoxic regions can be abrogated by knocking out HIF-1α ^77^. Furthermore, the mammalian target of rapamycin (mTOR) signaling pathway is involved in metabolic symbiosis during anti-angiogenic therapy, and the addition of rapamycin, an inhibitor of mTOR, can block this metabolism shift ^77^. Another study in a renal cell carcinoma patient-derived xenograft model showed that the metabolic symbiosis phenotype is involved in anti-angiogenic resistance and can be halted by blocking mTOR signaling ^78^. Inhibition of the upstream AKT/mTOR pathway can also sensitize renal cancer cells to multi-kinase inhibitor regorafenib ^79^.

Mitochondria contribute to the major part of oxygen consumption and have been found to influence cell signaling by producing reactive oxygen species (ROS) and metabolites ^80^. Tie-2 receptors and one of the ligands, Ang-1, are related to the activation of ROS and angiogenic response. Mitochondrial ROS can be triggered by Ang1/Tie2 signaling, and the released ROS can mediate the Ang1/Tie2 pathway and pro-angiogenic response ^81^. In breast and lung cancer models, a multi-kinase inhibitor could induce hypoxia-mediated tumor glycolysis and switch it to long-term reliance on mitochondrial respiration ^82^. Mutation in two mitochondrial genes—oxoglutarate dehydrogenase ( *OGDH*) and lipoic acid synthase ( *LIAS*)—can stabilize HIF-1α in a non-hydroxylated form, and the depletion of OGDH or LIAS leads to increased HIF-1α ^83^. The induction of metabolic symbiosis in response to anti-angiogenic therapy enables tumor cells to circumvent the anti-tumor effects of therapeutic agents by using cell survival pathways. It is clear that mitochondria, as the primary energy factory, are highly involved in hypoxia responses and help tumor cells survive anti-angiogenic therapy.

### Invasion and metastasis

Many studies have shown that anti-angiogenic therapy promotes tumor invasion and metastasis, which might be triggered by an anti-angiogenic therapy-associated increase in tumor hypoxia ^49, 51, 52, 71, 84^. The transcription of HIF-regulated genes is in control of diverse steps of tumor invasion and metastasis, including EMT, activation of MET signaling, recruitment of stromal cells, VM, and vessel co-option. It is reported that a triple-negative breast cancer mouse model exhibits increased MMP2 levels after discontinuation of sunitinib and VM channels were also observed accompanied by reduced endothelium-dependent vessel development ^61^. Data from patient samples revealed that the development of VM has a positive correlation with high expression of HIF-1α, MMP2, VE-cadherin, and CD31 ^61^. In breast cancer, right open reading frame (RIO) kinase 3, a conserved protein of atypical serine/threonine protein kinases, is involved in promoting hypoxia-induced invasion and metastasis via maintaining actin cytoskeletal organization ^85^. Hypoxia induces circadian clock gene period 2 ( *PER2*) degradation and enhances invasion and activation of EMT genes ( *TWIST1*, *SLUG*, and *SNAIL*) in breast cancer ^86^. Two independent signaling loops have been clarified to be involved in hypoxia-stimulated breast cancer invasion and metastasis: (i) in C-X-C chemokine ligand 16 (CXCL16) signaling, cancer cells secrete CXCL16, which binds to C-X-C chemokine receptor type 6 (CXCR6) on mesenchymal stem cells (MSCs), and in turn MSCs secrete CXCL10, which binds to CXCR3 on cancer cells, and (ii) MSCs secrete chemokine ligand 5 (CCL5), which binds to C-C chemokine receptor type 5 (CCR5) on cancer cells, and cancer cells release colony-stimulating factor 1 (CSF1), which binds to CSF1R on MSCs ^87^. These two pathways are both dependent on HIF activity and promote the recruitment of tumor-associated macrophages (TAMs) and myeloid-derived suppressor cells (MDSCs) ^87^. Hence, hypoxia induced by anti-angiogenic therapy could promote tumor invasion by accelerating the development of VM, vessel co-option, and EMT phenotypes. As mentioned above, the HIF-1α–ZEB2–ephrinB2 axis is an important regulatory pathway in promoting tumor invasiveness and evasive resistance in glioma during bevacizumab treatment ^49^. Anti-angiogenic agents induced the accumulation of Tie2-expressing macrophages (TEMs) at the invasive front of glioma tumor and TEMs can enhance the invasiveness of glioma tumor by secreting MMPs ^88^. Altogether, these studies offer opportunities for overcoming invasion and metastasis resulting from anti-angiogenic therapy.

## The role of stromal cells in resistance to anti-angiogenic therapy

TME is composed of resident (ECs and fibroblasts) and infiltrating (lymphocytes and macrophages) cells, extracellular matrix (collagen and fibronectin), and released molecules (cytokines, chemokines, antibodies, proteases, and angiogenic factors). One possible mechanism for resistance to anti-angiogenic therapy might be due to the recruitment of stromal cells. We and others have studied the complex interplay between ECs, platelets, pericytes, cancer-associated fibroblasts (CAFs), and white blood cells in the context of response to anti-angiogenic therapy ^11, 89^.

### Endothelial cells

The crosstalk between ECs and other stromal cells plays a critical role in response to anti-angiogenic therapy. Ang/Tie signaling is one of the central pathways that controls blood vessel growth, cell–cell interactions, and anti-angiogenic resistance. Ang2-regulated interactions between ECs and pericytes/myeloid cells are among the resistance mechanisms to anti-angiogenic therapy. For instance, bevacizumab could enhance Ang2/Tie2 signaling in ECs and upregulate Ang2 expression, which leads to reduced pericyte coverage and increased macrophage infiltration in brain cancer ^90^. Heterogeneity of tumor ECs (TECs) might also contribute to resistance to anti-angiogenic therapy. TECs are different from normal ECs in many ways, including cell proliferation, migration, gene expression profile, and response to therapy. TECs are resistant to some chemotherapeutic drugs such as vincristine, 5-fluorouracil, and paclitaxel owing to the upregulation of drug resistance-associated genes ^91^. CXCR4 is selectively expressed in TECs, and CXCR4 ^+^ TECs are related to poor outcome in patients with HCC. Functional studies revealed that CXCR4 is enriched in HCC angiogenic tip cells and overexpression of CXCR4 in ECs could stimulate vessel formation and sprouting *in vivo* and *in vitro*, implicating an important role for CXCR4 ^+^ TECs in angiogenesis ^92^. Furthermore, sorafenib shows higher anti-tumor efficacy in HCC tumors with high CXCR4 ^+^ expression ^92^. Interestingly, the recruitment of collagen type I ^+^/CXCR4 ^+^ fibrocyte-like cells can contribute to acquired resistance to bevacizumab ^93^. The activation of CXCR4, mediated mainly by CXCL12 (ligand for CXCR4), is induced by HIF-1α in hypoxic conditions ^93^. Similarly, a CXCR4 antagonist could interfere with neovascularization by preventing the interaction of CXCR4 ^+^ bone marrow-derived myeloid cells (BMDCs) and SDF-1α ^94^. Collectively, these findings reveal potential markers for predicting response to anti-angiogenic therapy. Although the pathways by which TECs mediate resistance to anti-angiogenic therapy are not fully understood, such research holds promise for enhancing anti-angiogenic therapy.

### Tumor-associated macrophages

BMDCs play a crucial role in the progression of angiogenesis and resistance to anti-angiogenic therapy. Many studies have shown that recruitment of BMDCs in GBM can cause resistance to vatalanib treatment and correspondingly the depletion of BMDCs can potentiate the effects of vatalanib ^95– 97^. Hypoxia-regulated neuropilin-1 (Nrp1), a marker of pro-angiogenic macrophages, can regulate the infiltration of TAMs into tumor hypoxic regions, and loss of Nrp1 in macrophages reduced angiogenesis and tumor growth ^98^. Future studies are needed to determine whether Nrp1 contributes to the acquisition of resistance to angiogenesis inhibitors and the underlying mechanisms. Another study revealed that the recruitment of TAMs in bevacizumab-resistant xenografts is caused by proliferation of differentiated macrophages and macrophage polarization and increases in numbers of pro-angiogenic macrophages ^99^. Bevacizumab can reduce macrophage inhibitory factor (MIF) expression at the edge of the tumor during early treatment while the loss of MIF leads to increased proliferation of TAMs in this area and eventual reprogramming into pro-angiogenic macrophages, even while treatment is continued ^99^. Pro-angiogenic macrophages promote tumor growth and invasion by secreting factors (for example, VEGFA, tumor necrosis factor alpha [TNFα], and interleukin-2 [IL-2]), eventually resulting in resistance to bevacizumab. In addition, hypoxia-induced chemokines (CXCL) and their receptors (CXCLR) have been shown to enhance the recruitment of TAMs and contribute to the emergence of therapeutic resistance ^89^.

### Tie2-expressing macrophages

TEMs are a subpopulation of TAMs. Crosstalk between TEMs and other stromal cells can enhance pro-angiogenic effects. For example, the interaction of TEMs and Tie2 ^+^ endothelial tip cells can promote vascular anastomoses during embryonic angiogenesis, and the blockade of the Ang2/Tie2 pathway in mannose receptor (MRC1)-expressing TEMs can impede angiogenesis ^100, 101^. In the RIP1-Tag2 pancreatic neuroendocrine tumor model, VEGFR2 inhibition upregulates Ang2 levels and enhances infiltration of TEMs. It can be halted by applying dual inhibitors of Ang2 and VEGFR2, which indicates that the adaptive enforcement of Ang2/Tie2 signaling induced by VEGFR inhibition may contribute to resistance ^102^. Similarly, in a murine GBM mouse model, blockade of Ang2 and VEGF resulted in decreased vascular permeability, decreased TEMs, and increased pericyte coverage and intratumoral T lymphocytes. Ang2 comes mainly from ECs and can mediate the interaction of ECs and myeloid cells ^90^. A strategy of dual blockade of Ang2 and VEGFR has shown better vascular normalization and TAM-phenotype shift than single-agent therapy ^103^. However, a recent phase II study showed that trebananib, an Ang1/2 inhibitor, was not effective as monotherapy in recurrent glioblastoma and did not improve outcomes in combination with bevacizumab. It is possible that such a dual inhibition strategy would be more effective in other cancer types ^104^.

### Pericytes

Pericytes play an important role in angiogenesis and vessel maturation, although the specific mechanisms involved are only partially elucidated ^105^. Angiogenic sprouting of ECs is facilitated by the detachment of pericytes, and vessel maturation requires the recruitment of supporting pericytes. The interactions between pericytes and ECs mediated by Ang/Tie signaling are a crucial step for blood vessel stabilization ^106, 107^. A previous study demonstrated a bidirectional, reciprocal relationship between ECs and pericytes via Ang/Tie2 signaling, as pericytes can also express functional Tie2 receptor ^108^. Several studies have focused on elucidating the mechanisms of pericytes in vessel stabilization or dysfunction ^106, 109– 111^. In terms of whether targeting pericytes could alleviate resistance to anti-angiogenic therapy, there is variability in the preclinical data. Recruitment of pericytes to tumor blood vessels is mediated by PDGF signaling and dual targeting of VEGF-mediated angiogenesis, and PDGF-mediated pericyte recruitment was found to be more effective than targeting VEGF-mediated angiogenesis alone in a RIP1-Tag2 mouse model ^112^. However, a subsequent study demonstrated that the absence of pericytes in tumors does not enhance the efficacy of anti-VEGF therapy in pericyte-deficient *pdgfb ^ret/ret^* mouse models ^113^.

Several pericyte-targeted therapies (by targeting PDGFR, VEGFR, and Tie2) are aimed at reducing tumor angiogenesis by blocking EC–pericyte interactions ^114^. For example, trebananib (Ang2 inhibitor) and nintedanib (VEGFR/FGFR/PDGFR inhibitor) show clinical benefits for patients with advanced ovarian cancer when combined with chemotherapy ( Table 1). One study in patients with breast cancer has shown that an increased pericyte-covered microvascular density (MVD), a marker of vascular normalization, is associated with improved pathologic response during post-bevacizumab monotherapy ^115^. Some studies suggest that pericytes can be used for predicting response to anti-angiogenic therapy. A retrospective study has revealed that *PDGFR-β* which is related to pericyte maturation can predict bevacizumab efficacy in patients with colon cancer ^116^. Similarly, it was shown that, in triple-negative breast cancer, tumors with high PDGFRβ ^+^/low desmin ^+^ pericytes coverage were more responsive to anti-angiogenic therapy ^117^. However, elucidating the mechanisms of pericytes mediating resistance to anti-angiogenic therapy still requires additional work.

### Endothelial progenitor cells

Endothelial progenitor cells (EPCs) have been shown to promote the angiogenic switch in solid tumors, and the recruitment of EPCs from bone marrow can directly contribute to tumor development and colonization. The recruitment of EPCs is induced primarily by hypoxia, and their contribution to tumor vasculature might stimulate resistance to anti-VEGF therapies ^89, 118^. Various factors are involved in the activation and mobilization of EPCs, including HIF-1α, VEGF, SDF1, MMPs, and membrane-bound kit ligand (mbKitL) ^89^. A recent study showed that interactions between EPCs and ECs are independent of hypoxia and the pro-angiogenic effects of EPCs on ECs were not completely dependent on the presence of VEGFA ^119^. Thus, VEGF-independent activation of EPCs could counteract the effects of anti-VEGF therapy and result in resistance. The circulating EPC frequency and the level of phospho-ERK in EPCs are a potential biomarker of sorafenib efficacy ^120^.

### Myeloid-derived suppressor cells

MDSCs can promote metastasis in animal models and cancer patients by supporting tumor cell survival, angiogenesis, invasion, and metastasis ^121, 122^. The role of immature myeloid cells/MDSCs in mediating resistance to anti-angiogenic therapy was first reported in preclinical studies by Shojaei *et al*. ^123, 124^. It has been suggested that MDSCs cause tumor resistance to anti-angiogenic therapy in several different ways, including (i) enhanced recruitment and infiltration of MDSCs, (ii) altered gene expression, (iii) phenotype differentiation, and (iv) activation of alternative growth factors ^89, 125– 127^. A persistence of intratumoral MDSCs is observed in sunitinib-resistant mouse models and may be related to local expression of granulocyte macrophage colony-stimulating factor (GM-CSF) and activation of STAT5 ^126^. This is confirmed in patients with sunitinib-treated tumors that show persistent elevation in MDSCs with increasing levels of pro-angiogenic factors such as MMPs and IL-8 ^126^. However, the detailed pathways of MDSC-mediated resistance to anti-angiogenic therapy and their clinical relevance are not fully understood.

### Platelets

As a well-known mediator for thrombosis and hemostasis, platelets have been recognized as a critical component of angiogenesis, metastasis, and tumor progression via releasing pro-angiogenic and anti-angiogenic factors ^128, 129^. Although the functional role of platelets in regulating angiogenesis has been reviewed ^130^, little is known about the role of platelets in response to anti-angiogenic therapy. Platelets might mediate resistance to anti-angiogenic therapy by secreting various growth factors and cytokines, interaction with EPCs and pericytes, uptaking anti-VEGF drugs, and promoting tumor invasion and metastasis ^89^. Platelet contents such as PDGF, FGF, angiostatin, and insulin-like growth factor (IGF) contribute to the development of tumors by interacting with myeloid cells or stimulating angiogenic factors ^131, 132^. A recent study demonstrated that platelet releasate exhibits a powerful pro-angiogenic effect on GBM-derived ECs and contains a high level of VEGF in patients with GBM as compared with normal controls ^133^.

### Other mechanisms

CAFs play a critical role in the TME. The expression of SDF1 and PDGF-C in CAFs has been reported in drug-resistant tumors ^89^. Crawford *et al*. first reported a role of CAFs in mediating resistance to anti-angiogenic therapy in a preclinical study ^134^. A recent study reported that CD44 ^+^ CAFs are increased following treatment with angiogenesis inhibitors and contribute to the maintenance of cancer stem cell populations, which associate with drug resistance ^135^. Marrow-derived fibrocyte-like cells with expression of alpha-1 type I collagen and CXCR4 have been demonstrated to contribute to acquired resistance to bevacizumab by producing FGF2 ^93^. Anti-angiogenic therapy has been shown to modulate and enhance the immune response in patients with cancer. For example, decreased regulatory T (Treg) cells have been noted during bevacizumab treatment in patients with mCRC and GBM ^136^. Recent studies found that bevacizumab could increase CD4 ^+^ lymphopenia, which is associated with poor survival in GBM patients and immune response suppression ^136^. However, another study showed that bevacizumab did not change the number, proliferation, or activation status in T-cell subsets within tumors but rather increased the percentage of M1/pro-inflammatory-polarized anti-tumor TAMs ^137^. A similar study showed that bevacizumab did not increase circulating suppressive MDSCs (lineage–HLADR–CD11b ^+^CD33 ^+^) but can increase the circulating concentration of soluble VEGFA ^136^.

## Targeting tumor microenvironment to overcome therapeutic resistance

The compensatory mechanisms such as the expression of other pro-angiogenic factors, hypoxia, and the crosstalk between tumor and stromal cells can be a new target to overcome resistance to anti-angiogenesis therapy. The emerging strategies targeting TME include new specific inhibitors, combined pathway inhibitors, multi-targeting strategies, and new approaches for drug delivery.

### New inhibitors

Several specific antagonists of VEGF(R) have been investigated in recent years. iVR1, a new inhibitor of VEGFR1, could inhibit colorectal cancer growth, macrophage migration, and monocyte mobilization by blocking the phosphorylation of VEGFR1 ^138^. Meanwhile, new antibodies are being investigated to target different molecules except for VEGF. For example, monoclonal antibodies against endoglin (CD105), a protein receptor of the transforming growth factor-beta (TGF-β) superfamily, showed a promising anti-vascular effect ^139^. A single-chain fragment of anti-human Ang2 has been shown to inhibit tumor growth, reduce vascular permeability, and extend survival in a bevacizumab-treatment GBM mouse model ^140^. Delta-like ligand 4-NOTCH1 signaling has been demonstrated to mediate tumor resistance to anti-VEGF therapy in preclinical models by activating multiple pathways ^141^. In preclinical ovarian cancer models, we have shown that dual targeting of DLL4 and VEGF exhibits superior anti-tumor effects ^142^. Two humanized DLL4 antibodies—enoticumab (REGN421) and demcizumab (OMP-21M18)—have shown preliminary anti-tumor activity in ovarian cancer and other solid tumors in phase I studies ^143, 144^. A bispecific DLL4/VEGF (OMP-305B83) antibody is also in phase Ib investigation with paclitaxel in ovarian cancer (ClinicalTrials.gov identifier: NCT03030287).

### Combined pathway inhibitors

As hypoxia plays a critical role in cancer progression, metastasis, and resistance to anti-angiogenic therapy, the development of hypoxia inhibitors could be a powerful approach for cancer treatment. A novel small molecule named saltern amide A (SA) can inhibit HIF-1α in various human cancer cells. SA suppressed PI3K/AKT/mTOR, p42/44 MAPK, and STAT3 signaling ^145^. Results from a phase I trial of bortezomib (a HIF-1α transcriptional activity suppressor) plus bevacizumab demonstrated clinical activity in patients with various tumors, including renal cell, breast, and ovarian/fallopian tube cancers ^146^. A phase I study in a combination with bevacizumab and EZN-2208 (PEGylated SN-38), another HIF-1α transcriptional activity inhibitor, showed acceptable toxicity in patients with refractory solid tumors. However, owing to the limited number of patients, the results did not demonstrate a conclusive effect of EZN-2208 on the activity of HIF-1α ^147^. The combination of an HDAC inhibitor and anti-angiogenic agents can downregulate HIF-1α and VEGF expression ^13^. Similarly, another study showed that the combination of metronomic topotecan and pazopanib can improve treatment response compared with the single drugs alone in metastatic triple-negative breast cancer ^106^. The potential mechanism might be related to the downregulation of HIF-1α induced by low-dose, continuous topotecan treatment ^148^. HIF-1α dimerization inhibitor acriflavine can enhance the anti-tumor efficacy of sunitinib by inhibiting VEGF and TGF-β expression and the accumulation of MDSCs in the spleen ^149^.

Given the adaptation of the immune cells during anti-angiogenic therapy, combination of anti-angiogenic agents with immune drugs is being investigated. Immune checkpoint inhibitors such as ipilimumab, nivolumab, and pembrolizumab show promising anti-tumor effects by augmenting anti-tumor immune responses ^150, 151^. Programmed cell death-1 (PD-1) receptor, the negative immune checkpoint regulator, and its ligand, PD-L1, which can suppress immune response, have been shown to be upregulated during anti-angiogenic therapy ^152, 153^. Thus, it provides feasible approaches to enhance response to anti-angiogenic therapy by adding immune checkpoint inhibitors. Several studies have reported that adding immune checkpoint agents shows improved clinical benefit compared with anti-angiogenic monotherapy ^154^. A2V, a novel bevacizumab-based bispecific human IgG1 antibody that targets Ang2 and VEGFA, has been found to promote anti-tumor immunity by activating tumor-infiltrating CD8 ^+^ T cells, increasing tumor antigen presentation, and enhancing perivascular T-cell accumulation ^152^. Also, A2V can increase PD-L1 expression via interferon-gamma (IFNγ) signaling and combining PD-1 blockade and A2V can improve the anti-tumor activity in certain tumor models ^152^. The enhanced effect of adding PD-L1 inhibitor to anti-angiogenic therapy is dependent on the induction of high endothelial venules, which can facilitate lymphocyte infiltration via lymphotoxin β receptor signaling ^153^. A series of clinical trials of combined anti-angiogenic therapy with immune checkpoint therapy is ongoing ^154^.

As VEGF-independent angiogenesis pathways can contribute to resistance to anti-VEGF therapy, the combination treatment of chemotherapeutic agents and anti-VEGF therapy may overcome such drug resistance. A study of the combination of vascular disrupting agents (VDAs) and sunitinib was found to result in improved treatment efficacy in a colorectal liver metastasis mouse model by reducing tumor proliferation and vasculature and increasing tumor apoptosis ^155^. Similarly, a phase II trial showed that the addition of VDAs to bevacizumab can extend PFS duration in patients with recurrent ovarian cancer ^30^. The combination of VEGF/VEGFR inhibitors with anti-invasive drugs or vessel co-option inhibitors may provide another possibility to overcome resistance. A recent phase I study in patients with recurrent GBM tested the combined effect of the VEGFR inhibitor cediranib with the invasion inhibitor cilengitide. Although no increased toxicities were observed in the combination treatment of cediranib and cilengitide, no survival benefit was shown ^156^. However, recent preclinical work has shown that cilengitide can in fact promote tumor invasion, tumor growth, and tumor angiogenesis and therefore may not be the ideal drug to combine with anti-angiogenic therapy in the clinic ^157, 158^. Despite the promising future of combining anti-angiogenic therapy with anti-invasive agents or vessel co-option inhibitors, successful clinical translation has yet to be achieved.

### Multi-targeting strategy

Based on the compensatory responses to anti-VEGF therapy, combining treatments that target multiple angiogenic signals could be important. Preclinical models showed that the combination of multi-tyrosine kinase inhibitors lenvatinib (VEGFR, FGFR, and RET inhibitor) and golvatinib (E7050; c-Met, Tie2, and EphB4 inhibitor) could inhibit the development of pericytes and infiltration of TEMs in thyroid and endometrial cancer models ^159^. Apart from VEGF/VEGFR inhibitors, targeting PDGF/PDGFR signaling can also improve the efficacy of current therapy and reduce tumor growth, invasion, and metastasis ^160^. Nonetheless, one study showed that the depletion of pericytes by imatinib and sunitinib not only can reduce tumor growth but also can increase metastasis and EMT progression ^161^. Another study revealed that depletion of PDGFRβ ^+^ pericytes at early stages of tumor progression reduced metastasis but enhanced metastasis at later stages; further study implicated Ang2 as a key mediator of the metastatic phenotype ^162^. Notably, the increased metastasis induced by pericyte depletion can be limited by additional MET or Ang2 inhibitors, which may provide a new and efficient strategy to suppress tumor growth while minimizing the risk of metastasis ^161, 162^. A heparin-derived angiogenesis inhibitor, LHT7, targeting FGF2 and PDGF-β, could inhibit the maturation of endothelium and can serve as a potential drug together with VEGF inhibitors to overcome resistance ^163^. Further study indicated that the combination of LHT7 and a selective cyclooxygenase-2 (COX2) inhibitor (celecoxib) showed a stronger therapeutic effect than anti-angiogenic drugs alone ^164^. COX2 has been reported to counteract the efficacy of anti-angiogenic agents ^164^. Lucitanib (a multi-target inhibitor of VEGFR1 to 3, PDGFRα/β, and FGFR1 to 3) has demonstrated activity in phase I/II clinical testing in patients with breast cancer ^165^. Another novel method to overcome resistance to bevacizumab therapy is combining VEGF inhibitors with pericyte-targeted drugs (mostly inhibitors of Ang or PDGFRβ). Ang2 and the VEGFA inhibitor A2V exert anti-tumor effects in a variety of ways, including impairing tumor angiogenesis, reducing metastasis, and increasing the infiltration of pro-inflammatory macrophages ^137, 166^. In a xenograft model of ovarian cancer, dual targeting of VEGF and Ang has been shown to result in greater inhibition of tumor angiogenesis and metastasis than monotherapy with either VEGF or Ang inhibitors ^167^. Another study showed that VEGF inhibitor and Ang2 inhibitor can potentially reduce resistance to anti-angiogenic therapy ^90^. Furthermore, imatinib could inhibit PDGFR ^+^ pericyte-like cells and disrupt tumor vascular integrity as well as EC survival ^168^. While trebananib (a first-in-class peptibody targeting Ang2) exhibited clinical benefit in patients with ovarian cancer, it was ineffective as monotherapy and did not enhance the effect of bevacizumab in patients with recurrent glioblastoma ^104, 169^. As first-line therapy, brivanib (a dual inhibitor of VEGFR and FGFR) had a similar anti-tumor effect but was less well tolerated compared with sorafenib in a phase III study ^170^. Another phase III study showed that brivanib as second-line therapy did not result in improved outcomes of HCC patients who did not respond to sorafenib ^171^. Additional work is needed to understand the true efficacy of multi-targeted therapy in different cancer types.

### Drug delivery

Nanoparticles can be designed with specific target proteins to deliver drugs into target cells. New sorafenib-loaded CXCR4-targeted nanoparticles have been designed to treat HCC. The results of *in vitro* and *in vivo* studies show that it can reduce the infiltration of TAMs and enhance anti-angiogenic effects. Nanoparticles designed to deliver sorafenib into tumors efficiently could be an innovative approach to overcome drug resistance ^172^.

## Conclusions

Although mechanistic links between TME and anti-angiogenic therapy have been studied, the overall mechanisms of resistance to anti-angiogenic therapy require additional work. The combination of VEGF(R) inhibitors and other pathway inhibitors, including hypoxia inhibitors or immune checkpoint inhibitors, is being evaluated in various clinical trials. Unfortunately, reliable biomarkers for predicting response or the emergence of resistance have not been identified. It is likely that combination treatments will be required for overcoming drug resistance and prolonging patient survival. In summary, anti-angiogenesis therapies remain a highly effective avenue for cancer therapy. Understanding the mechanisms of adaptive resistance will allow an improved understanding of the complex underlying biology and holds tremendous potential for innovative drug development.

## Abbreviations

Ang, angiopoietin; BMDC, bone marrow-derived cell; CA, carbonic anhydrase; CAF, cancer-associated fibroblast; COX2, cyclooxygenase-2; CSF1, colony-stimulating factor 1; CXCL, C-X-C chemokine ligand; CXCR, C-X-C chemokine receptor; EC, endothelial cell; EMT, epithelial-to-mesenchymal transition; EPC, endothelial progenitor cell; FAK, focal adhesion kinase; FGF, fibroblast growth factor; FGFR, fibroblast growth factor receptor; FIH-1, factor inhibiting 1; GBM, glioblastoma multiforme; HCC, hepatocellular carcinoma; HDAC, histone deacetylase; HIF, hypoxia-inducible factor; IL, interleukin; mCRC, metastatic colorectal cancer; MAPK, mitogen-activated protein kinase; MDSC, myeloid-derived suppressor cell; MIF, macrophage inhibitory factor; MMP, matrix metalloproteinase; MSC, mesenchymal stem cell; mTOR, mammalian target of rapamycin; NAC1, nucleus accumbens-associated protein-1; Nrp1, neuropilin-1; OGDH, oxoglutarate dehydrogenase; OS, overall survival; PD-1, programmed cell death-1; PDGF, platelet-derived growth factor; PECAM, platelet endothelial cell adhesion molecule; PFS, progression-free survival; PHD, prolyl hydroxylase domain; ROS, reactive oxygen species; SA, saltern amide A; SDF1, stromal cell-derived factor 1; TAM, tumor-associated macrophage; TEC, tumor endothelial cell; TEM, Tie2-expressing macrophage; TGF-β, transforming growth factor-beta; TME, tumor microenvironment; VDA, vascular disrupting agent; VE-cadherin, vascular endothelial cadherin; VEGF/R, vascular endothelial growth factor/receptor; VM, vasculogenic mimicry
